# Cryptic papulonodular eruption with fever and vision loss

**DOI:** 10.1016/j.jdcr.2024.04.020

**Published:** 2024-04-23

**Authors:** Kostandin Valle, Daniel Tinker, Jamie Hanson, Duane Dilworth

**Affiliations:** a University of Missouri-Columbia School of Medicine, Columbia, Missouri; b Department of Dermatology, Saint Louis University, St Louis, Missouri; c Deluxe Dermatology, St Louis, Missouri

**Keywords:** case reports, granulomatous secondary syphilis, histopathological findings, secondary syphilis, sexually transmitted infection, treponema pallidum

## Patient history

**Fig 1:**
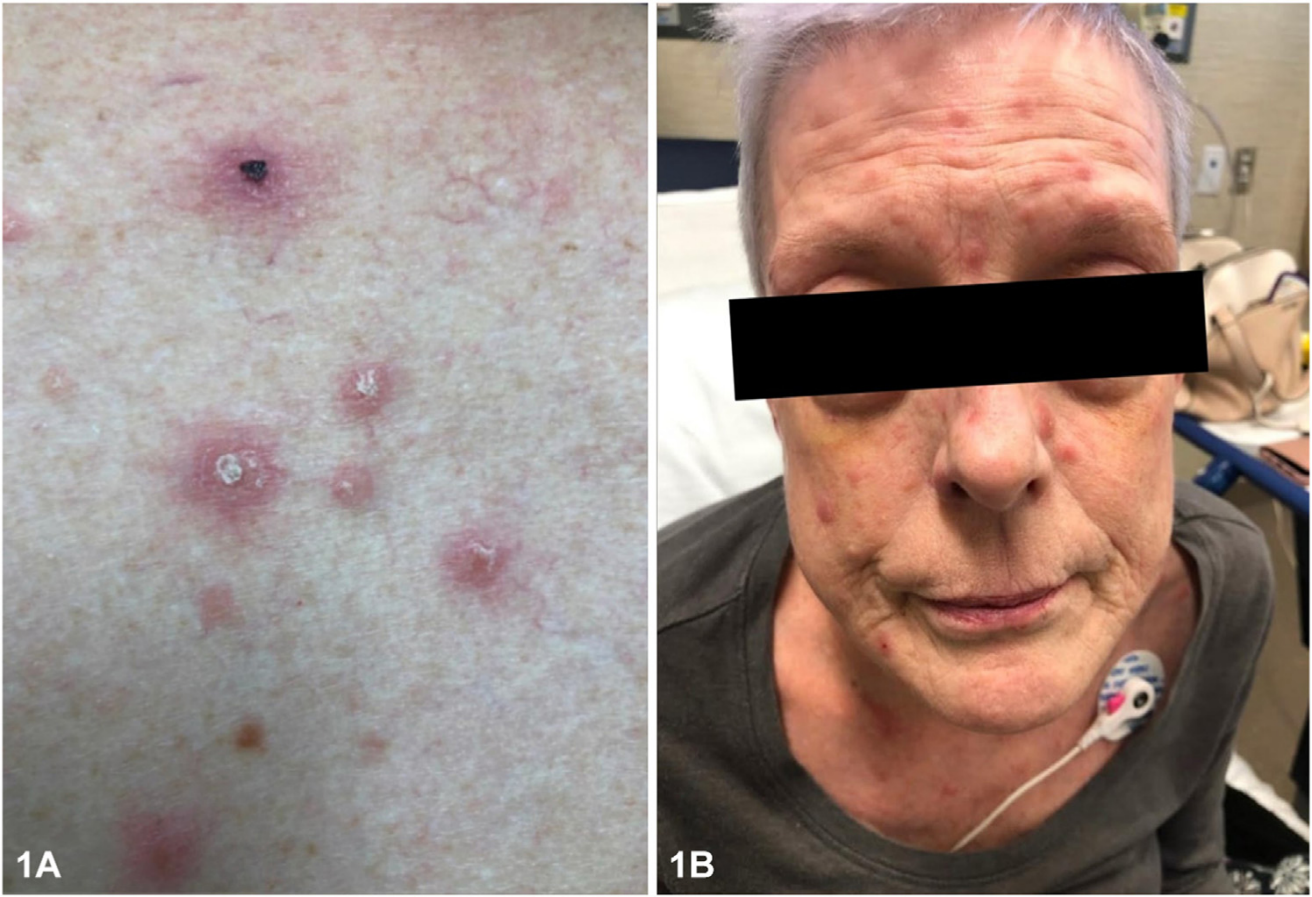

**Fig 2:**
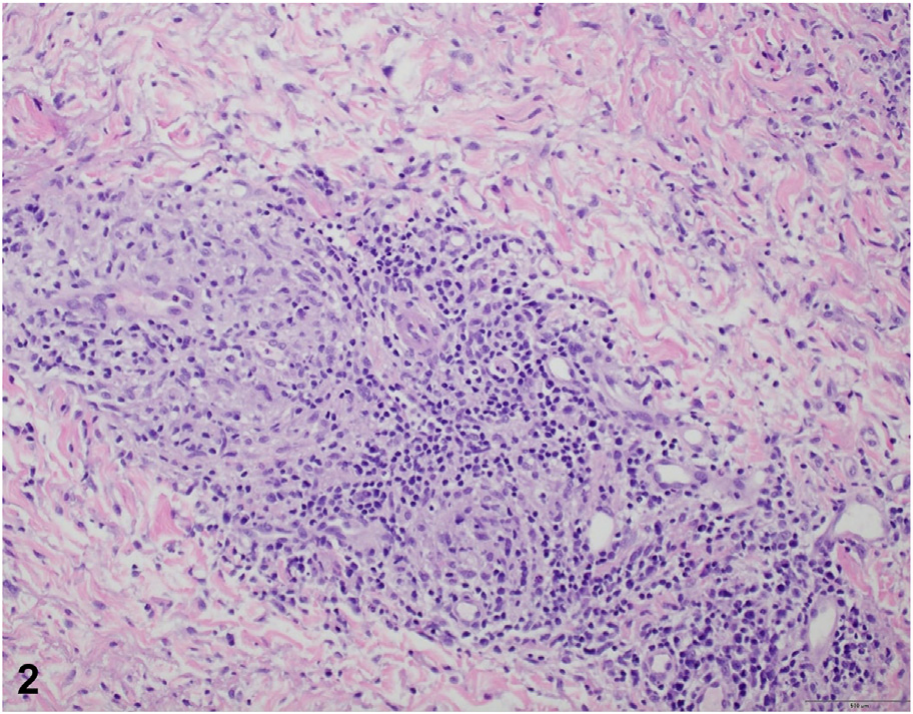

A 53-year-old Caucasian female presented with a 2-week history of a nonpruritic rash involving the abdomen, chest, back, and extremities as well as fever and an episode of sudden left eye vision loss. Physical examination revealed numerous 0.5 to 2.5 cm pink papulonodular lesions with central crusting and collarets of scale widely distributed on the face, chest, abdomen, back, and extremities. No lymphadenopathy or mucosal involvement was present. Two punch biopsies revealed granulomas with histiocytes, multinucleated giant cells lymphocytes, and plasma cells (Figs 1 and 2).

**Question 1: What is the diagnosis based on these clinicopathologic images?**

A. Primary cutaneous B-cell lymphoma (CBCL)
B. Sarcoidosis
C. Secondary syphilis
D. Disseminated histoplasmosis
E. Cutaneous tuberculosis

**Answers:**

A. CBCL — Incorrect. Indeed, primary CBCL may present as erythematous papules, nodules, plaques, and tumors on the face; however, the lesions are often more violaceous without central erosion and crusting. Histology would show diffuse or nodular dermal infiltrate of lymphocytic cells.
B. Sarcoidosis — Incorrect. While the patient’s fever, vision loss, and cutaneous eruption could be seen in sarcoidosis, histology would show epithelioid histiocytes forming well-demarcated granulomas that are “naked” without associated lymphoplasmacytic infiltrate.
C. Secondary syphilis — Correct. Because of its protean clinical manifestations, secondary syphilis has been suitably referred to as “the great mimicker”. Such pronounced ocular involvement is an unusual presentation in secondary syphilis,^1^ as is the histologic finding of predominant granulomatous, admixed with plasma cells.^2^, ^3^, ^4^ This case underscores the high index of suspicion one must have for syphilis because of its variability in presentation and the serious morbidity it possesses that can be easily prevented if recognized and treated early.^5^
D. Disseminated histoplasmosis — Incorrect. While disseminated histoplasmosis certainly could present similarly with numerous eroded and crusted papules on the face and trunk with associated systemic symptoms (eg fever), biopsy would demonstrate characteristic intracellular yeast forms surrounded by a rim of clearing within macrophages and giant cells.
E. Cutaneous tuberculosis — Incorrect. Cutaneous tuberculosis presentations are nonspecific with considerable clinical heterogeneity and may present similarly (eg, papulonecrotic tuberculoid or lichen scrofulosorum presentations). Histologically, a plasma cell dermal infiltrate could be seen; however, tuberculosis classically presents as granulomatous inflammation with central caseation.

**Question 2: What additional workup would confirm your suspected diagnosis?**

A. Interferon-γ release assay (eg, QuantiFERON TB Gold)
B. Injecting suspension of sarcoidal spleen into the skin of the patient and observing for sarcoid granuloma formation
C. Immunohistochemistry (IHC) staining for CD20, CD79a, BCL-2, BCL-6, and MUM-1
D. Histoplasma antigen testing of blood and urine
E. IHC and serological testing for *Treponema pallidum*

**Answers:**

**Fig 3:**
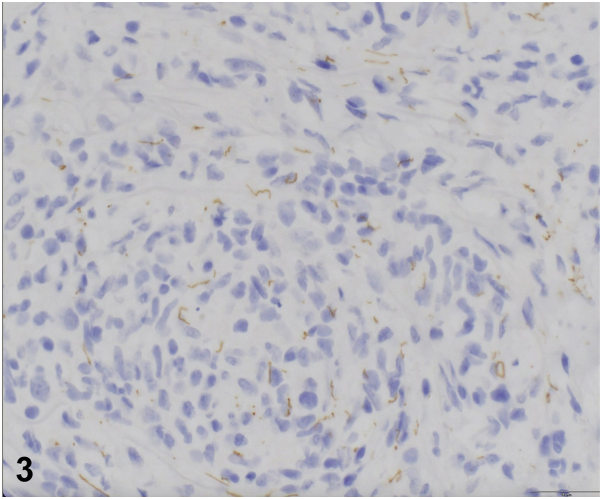

A. Interferon-γ release assay (eg, QuantiFERON TB Gold) — Incorrect. This would confirm the diagnosis of cutaneous tuberculosis but as described above, the histology is not consistent with tuberculosis.
B. Injecting suspension of sarcoidal spleen into the skin of the patient and observing for sarcoid granuloma formation — Incorrect. This describes the Kveim-Siltzbach test which is not routinely performed to diagnose sarcoidosis.
C. Immunohistochemistry (IHC) staining for CD20, CD79a, BCL-2, BCL-6, and MUM-1 — Incorrect. These IHC stains would help differentiate between the most common types of primary CBCL.
D. Histoplasma antigen testing of blood and urine — Incorrect. This would confirm the diagnosis of disseminated histoplasmosis but as described above, the histology is not consistent with histoplasmosis.
E. IHC and serological testing for *Treponema pallidum* — Correct. The most sensitive and specific way to confirm the suspected diagnosis of syphilis. The patient in this case had a reactive *T. pallidum* antibody (FTA-ABS) as well as a reactive rapid plasma reagin, titer of 1:28. IHC would show *T. pallidum* spirochetes, as were present in this patient (Fig 3).

**Question 3: Based on your diagnosis, what is your next step in management?**

A. Rifampin, isoniazid, pyrazinamide, and ethambutol and referral to infectious disease
B. Amphotericin B followed by itraconazole
C. Intralesional anti-CD20 monoclonal antibodies, such as rituximab and radiation therapy
D. Intramuscular benzathine penicillin, 2.4 million units
E. Oral glucocorticoids followed by a slow, prolonged taper

**Answers:**

A. Rifampin, isoniazid, pyrazinamide, and ethambutol and referral to infectious disease — Incorrect. This would be appropriate to treat cutaneous tuberculosis but not granulomatous secondary syphilis.
B. Amphotericin B followed by itraconazole — Incorrect. This would be appropriate to treat disseminated histoplasmosis but not granulomatous secondary syphilis.
C. Intralesional anti-CD20 monoclonal antibodies, such as rituximab and radiation therapy — Incorrect. This would be appropriate to treat CBCL but not granulomatous secondary syphilis.
D. Intramuscular benzathine penicillin, 2.4 million units — Correct. The best treatment for granulomatous secondary syphilis. Additionally, if ocular involvement is suspected to be due to *T. pallidum*, a lumbar puncture should be performed. If central nervous system or ocular syphilis is confirmed, the treatment would be 10 to 14 days of intravenous penicillin G.
E. Oral glucocorticoids followed by a slow, prolonged taper — Incorrect. This would be appropriate to treat systemic sarcoidosis but not granulomatous secondary syphilis.

## Conflicts of interest

Author Kostandin Valle and Drs Daniel Tinker and Jamie Hanson have no conflicts of interest to declare. Dr Dilworth is a speaker for Galderma Lilly, and Pfizer. He is also a consultant for Sanofi and an advisor for Bristol Meyers Squibb.

## References

[1] Furtado J.M., Simões M., Vasconcelos-Santos D. (2021). Ocular syphilis. Surv Ophthalmol.

[2] Flamm A., Parikh K., Xie Q., Kwon E.J., Elston D.M. (2015). Histologic features of secondary syphilis: a multicenter retrospective review. J Am Acad Dermatol.

[3] Rysgaard C., Alexander E., Swick B.L. (2014). Nodular secondary syphilis with associated granulomatous inflammation: case report and literature review. J Cutan Pathol.

[4] Lee T.-H., Yang T.-H., Chang Y.-S., Chang I.-J. (2020). Granulomatous secondary syphilis with a facial annular sarcoidosis-like clinical and histopathological pattern. Australas J Dermatol.

[5] National Academies of Sciences, Engineering, and Medicine (2021).

